# Successful treatment of a necrotizing fasciitis related skin defect using fish skin grafting in a patient with extensive psoriasis vulgaris

**DOI:** 10.1016/j.jdcr.2026.01.041

**Published:** 2026-01-31

**Authors:** Imad Al-naesan, Nina Frischhut, Wolfgang Schlosser, Dietmar Pixner

**Affiliations:** a Department of General Surgery, Hospital Reutte, Reutte, Austria; b Department of Dermatology, Venereology and Allergy, Medical University of Innsbruck, Innsbruck, Austria

**Keywords:** fish skin graft, histopathology, necrotizing fasciitis, psoriasis, skin graft

## Introduction

Necrotizing fasciitis (NF) is an infection of deep soft tissues that progressively destroys the muscle fascia and nearby subcutaneous fat and can occur at various sites once the skin barrier is breached.^1^ Although rare, NF is highly destructive and potentially lethal. While antibiotics and critical-care assessment are essential, the primary treatment is surgical debridement. After removing necrotic tissue, reconstruction of extensive soft-tissue defects is challenging. Reconstructive options may include skin flaps, split-thickness grafts, or free-tissue transfer.^2^ Recently, acellular fish skin grafts have emerged as a potentially cost-effective wound-healing modality with improved outcomes.^3^^,^^4^ We present a unique case of a psoriasis patient whose skin defect from NF was successfully covered using a fish skin graft.

## Case presentation

**Fig 1:**
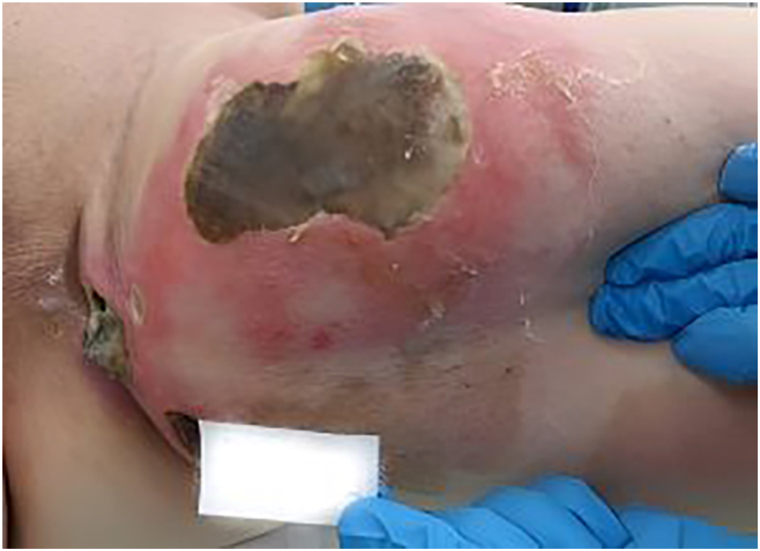
Initial presentation of the patient at our clinic with a pronounced, partly necrotizing soft-tissue infection of the skin on the left thigh.

**Fig 2:**
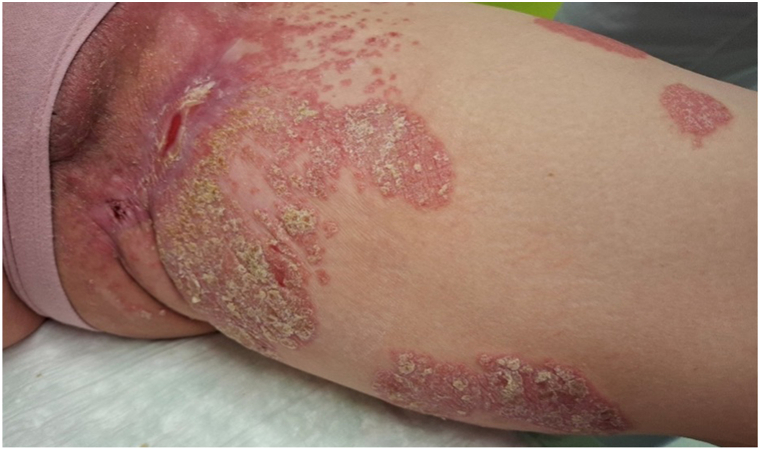
Presentation of our patient 8 weeks after surgical coverage of the skin defect with a fish-skin graft. The skin is nearly completely healed, with a visible, partially infiltrating distribution of psoriasis.

**Fig 3:**
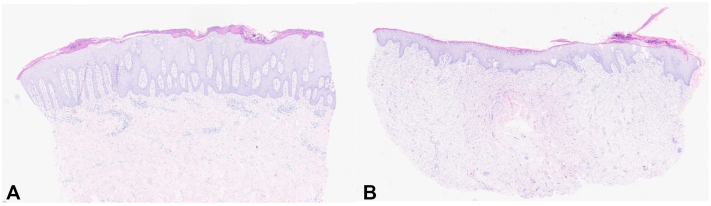
**A,** Histopathological examination (hematoxylin and eosin [H&E] stain) of the upper thigh reveals an elongated stratum corneum with parakeratosis and intracorneal neutrophilic inclusions, set in an acanthotic and papillomatous epidermis, typical for psoriasis. **B,** On H&E-stained biopsy from the margin of the fish transplant, the stratum corneum is thick and shows parakeratosis with serum crusts; underneath is an irregular, broadened epithelial layer with notable spongiosis, overall resembling a psoriasiform epidermitis.

A 46-year-old white woman with a history of psoriasis presented to the surgical department in Reutte with a markedly enlarging left thigh abscess and a severely deteriorated general condition (Fig 1). The infection originated from a minor skin injury that progressed rapidly. An immediate intraoperative abscess dissection and necrosectomy were performed. Empirical antibiotics (piperacillin/tazobactam and clindamycin) were started and after 1 day, this was adjusted to piperacillin/tazobactam, amoxicillin/clavulanate, and fosfomycin. During this period, delirium developed in the context of diabetes mellitus, hyperglycemia, metabolic acidosis, and electrolyte disturbances. Postoperatively, cardiac instability necessitated high dose catecholamines. Due to marked hemodynamic instability and worsening local findings within 24 hours, the patient was transferred to a tertiary center, the University Hospital of Innsbruck. In the intensive care unit, she received continued comprehensive critical care, including another debridement with a VAC system. In the meantime, intraoperative cultures yielded *Streptococcus gallolyticus* and *Candida glabrata*, prompting escalation to piperacillin/tazobactam plus linezolid with intravenous anidulafungin and topical nystatin. Histopathology showed florid phlegmonous and necrotizing inflammation without malignancy. Psoriasis was managed topically with topical betamethasone. After stabilization, care returned to the referring hospital in Reutte. VAC changes were repeated and autologous mesh skin grafting was attempted but failed. VAC therapy with a silver-impregnated sponge continued. After adequate wound granulation, a second coverage attempt using a fish-skin xenograft was planned 3 weeks later and was well tolerated, achieving satisfactory results. Wound checks were initially frequent and then weekly. After 8 weeks, a psoriatic plaque began to spread, appearing to extend onto the fish-skin graft macroscopically (Fig 2). Four 4 mm punch biopsies were taken (healthy skin, a psoriatic plaque on the outer thigh, the graft margin, the graft center). In the graft center, healing was characterized by secondary wound healing with marked fibrosis and an acanthotic, proliferative epithelial layer with parakeratosis, without evidence of psoriasis. The border biopsy showed spongiotic-psoriasiform epidermitis with signs of impetiginization, but no typical psoriasis pattern (Fig 3). Three months after xenograft implantation, the patient remains in remission with an intact skin and no psoriatic changes. Extension of psoriasis therapy had not been pursued due to the recent history but is now being planned.

## Discussion

NF can rapidly create extensive soft-tissue defects demanding prompt wound coverage. Fish-skin grafts, as xenogeneic skin substitutes, may be a promising option, particularly for patients like ours who have previously rejected autologous skin grafts. The graft’s natural collagen framework and omega-3–rich composition may support granulation and modulate local inflammation, contributing to a stable wound.^5^^,^^6^ Notably, psoriasis manifestations emerged near the transplant site, illustrating a Koebner phenomenon triggered by surgical intervention and local trauma, independent of the graft itself. However, challenges exist, including graft acceptance, potential for infection, and the possibility that xenografts may delay or alter re-epithelialization in some patients. Ongoing observation as well as systemic psoriasis treatment should be continued to minimize flares and to monitor disease activity for any potential impact on healing or graft durability. Despite a promising, outcome, further studies are needed to confirm our results.

## Conflicts of interest

None disclosed.
